# Monitoring Reaction Paths Using Vibrational Spectroscopies: The Case of the Dehydrogenation of Propane toward Propylene on Pd-Doped Cu(111) Surface

**DOI:** 10.3390/molecules23010126

**Published:** 2018-01-10

**Authors:** Wei Hu, Xinrui Cao

**Affiliations:** 1 School of Chemistry and Materials Science, University of Science and Technology of China, iChEM (Collaborative Innovation Center of Chemistry for Energy Materials), Hefei 230026, China; weihulp@ustc.edu.cn; 2 Department of Physics and Institute of Theoretical Physics and Astrophysics, Xiamen University, Xiamen 361005, China

**Keywords:** surface catalysis, surface-enhanced Raman spectroscopy, infrared spectroscopy, propane dehydrogenation

## Abstract

Monitoring reaction paths is not only a fundamental scientific issue but also helps us to understand and optimize the catalytic process. Infrared (IR) and Raman spectroscopies are powerful tools for detecting particular molecules or intermediate products as a result of their ability to provide the molecular “finger-print”. However, theoretical modeling for the vibrational spectra of molecular adsorbates on metallic surfaces is a long-standing challenge, because accurate descriptions of the electronic structure for both the metallic substrates and adsorbates are required. In the present work, we applied a quasi-analytical IR and Raman simulation method to monitor the dehydrogenation of propane towards propylene on a Pd-doped Cu(111) surface in real-time. Different Pd ensembles were used to construct the single-atom catalyst (SAC). We found that the number of sublayer Pd atoms could only affect the intensity of the peak rather than the peak position on the vibrational spectra. However, with the dehydrogenation reaction proceeding, both IR and Raman spectra were changed greatly, which indicates that every reaction step can be distinguished from the point of view of vibrational spectroscopies. Additionally, we found that the catalytic process, which starts from different initial states, shows different spectral profiles. The present results suggest that the vibrational spectroscopies obtained by the high-precision simulations pave the way for identifying different catalytic reaction paths.

## 1. Introduction

Studying reaction paths is not only a fundamental scientific issue, but it also helps in understanding and optimizing the catalytic process. Theoretically, transition state theory is usually employed to evaluate the activation energies; then the most likely reaction path can be determined accordingly. It is noted that the theoretical evaluations of activation energies are highly reliant on the simulation environments [1,2,3,4,5,6,7,8]. As a result, the relative values of activation energies rather than the absolute values are more useful in predicting reaction paths. Compared to the activation energies, spectroscopies usually provide more reliable information. Many technologies, such as nuclear magnetic resonance (NMR), X-ray photoelectron spectroscopy (XPS) and vibrational spectroscopies have been employed to detect specific intermediate products. On the basis of the detection of the intermediate product, the reaction path can be determined accordingly. Among these spectroscopic technologies, infrared (IR) and Raman spectra have advantages in detecting the intermediate products because of their in situ operability [1,2,3,4,9,10,11,12,13].

Up to now, there have been two approaches to model the adsorption of molecules on metallic surfaces and to simulate the corresponding IR and Raman spectra at the ab initio level: the cluster model and the periodic boundary condition (PBC) model. It is well known that the PBC model could provide a more reliable electronic structure of metallic substrates and nanoparticles compared to the cluster model. Therefore, many efforts have been made to develop IR and Raman simulation approaches using PBC models [14,15,16]. For example, Ding et al. developed a method that combines the geometrical optimization of the PBC model and Raman calculation of the cluster model [17]. They have tested the method by simulating the Raman spectra CO and ethylene adsorbed on Rh(111). Many efforts have also been made by our group in the previous works [18,19,20]. Our quasi-analytical method has also been applied in many specific systems, and the accuracy and stability have been tested [18,19,20].

Light olefins are important basic raw materials in the chemical industry. With their growing demand in the global chemical market, the catalytic production of olefins has attracted increased interest in the past decades [21]. It has been widely adopted that the dehydrogenation of light alkanes is a dominant commercial means to produce olefins. However, the highly endothermic process limits its industrial efficiency. Therefore, efficient catalysts are deeply desired. Recently, we designed a kind of single-atom catalyst (SAC) based on a Pd-doped Cu(111) surface. The theoretical simulations show that the embedded Pd atoms significantly improve the catalytic reactivity, whereas the presence of the Cu surface is beneficial for the diffusions of the detached H atoms. Therefore, we conclude that the high selectivity toward propylene dehydrogenation is realized on the Pd-doped Cu(111) surface. However, if these designed catalysts can be used in reality, how can we know the true reaction path from experiments at the in situ level?

In the present work, we attempt to apply our quasi-analytical method to systematically investigate the IR and Raman spectra of a whole reaction process. Specifically, the dehydrogenation of propane on Pd-doped Cu(111) is studied. By studying the changes in the IR and Raman spectra, the reaction paths are monitored in real-time. Most of all, different reaction paths are characterized, which provide us with a potential application of vibrational spectroscopies in tracing reaction paths.

## 2. Results

**Figure 1:**
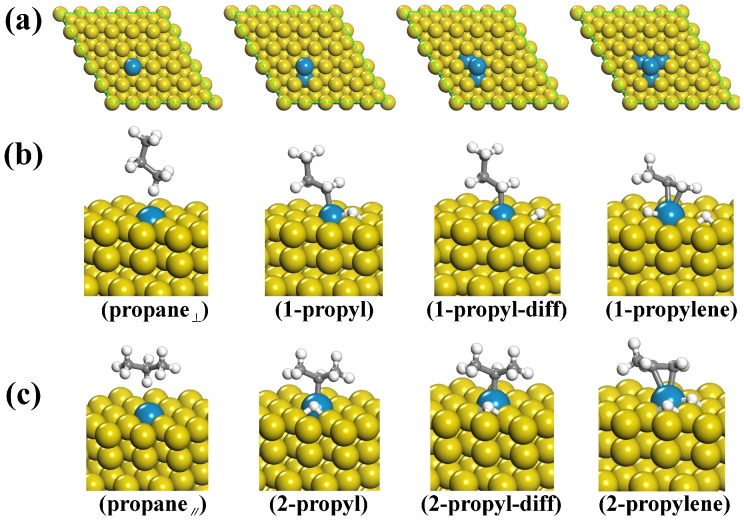
(**a**) Different Cu(111) surfaces with one to four doped Pd atoms; (**b**,**c**) Geometries of the initial reactants and intermediate and final products during the dehydrogenation process of the perpendicular and parallel adsorbed propane towards propylene.

To investigate the effect of multiple doping on the catalytic performance, we designed four different Pd-doped Cu(111) surfaces. As shown in Figure 1a, the number of doped Pd atoms ranged from one to four. It is noted that the most stable configuration of propane adsorbed on the surface has been proven to be a parallel state (shown in Figure 1c, labelled as propane_||_ hereafter), which agrees well with the previous works [22,23,24,25,26]. On the other hand, Figure 1b shows the second stable configuration, in which a perpendicular adsorption is adopted. Our previous studies show that both parallel and perpendicular adsorbed propane could be catalyzed to propylene [27]. To explore the transition states of the propane dehydrogenation process, we employed the widely used climbing-image nudged elastic band (CI-NEB) method [28]. Frequency calculations were also performed to characterize the obtained transition-state structures, and these intermediates have only one imaginary frequency according to our previous study [27]. The corresponding configurations for the initial reactants, transition states and final products for the first dehydrogenation reaction are shown in Figure 1b,c. We can see that the main difference between the two reaction paths is the order of the dehydrogenation process. For the parallel adsorbed configuration, the first dehydrogenation occurs on the methylene group, and the second occurs on the methyl group. However, for the perpendicular configuration, the order of the two dehydrogenation processes is reversed. For the sake of discussion, we label these stationary points as propane_||_ (propane_||_), 1-propyl (2-propyl), 1-propyl-diff (2-propyl-diff), and 1-propylene (2-propylene). In particular, 1-propyl-diff (2-propyl-diff) indicates an extra stable state in which the first detached proton is diffused. Transition-state calculations show that the first dehydrogenation process needs the highest activation energies and that the parallel configuration shows better catalytic performance than the perpendicular configuration. To be specific, the activation energies for the parallel configuration with the 1–4 Pd-doped Cu(111) surfaces were found to be reduced by 5.1, 6.2, 8.0 and 9.7 kcal/mol compared to the pure Cu(111) surface, resulting in 20–400 times increases in the reaction rates [27].

In Table 1, we tabulate the predicted energy barriers for the first C–H bond cleavage from the methyl and methylene groups of propane on the pure and Pd-doped Cu(111) surfaces. From Table 1, one can see that the calculated activation energies varied only a little for these representative reaction paths according to our previous study. Taking the propane dehydrogenation over the single Pd-doped Cu(111) surface for instance, the first C–H activation from the CH_3_ group was 32.8 kcal/mol, while the dehydrogenation of CH_3_ required an energy barrier of 31.6 kcal/mol. The energy difference between these two reaction paths was only 1.2 kcal/mol; this tiny difference makes it very hard to determine the reaction process in the true reaction from transition-state calculations. Therefore, it is highly desirable to find an appropriate way to determine the reaction paths. Because the IR and Raman spectroscopies can provide the molecular “finger-print”, investigating the spectral changes during the whole reaction process became an ideal solution for identifying the true reaction path.

**Figure 2:**
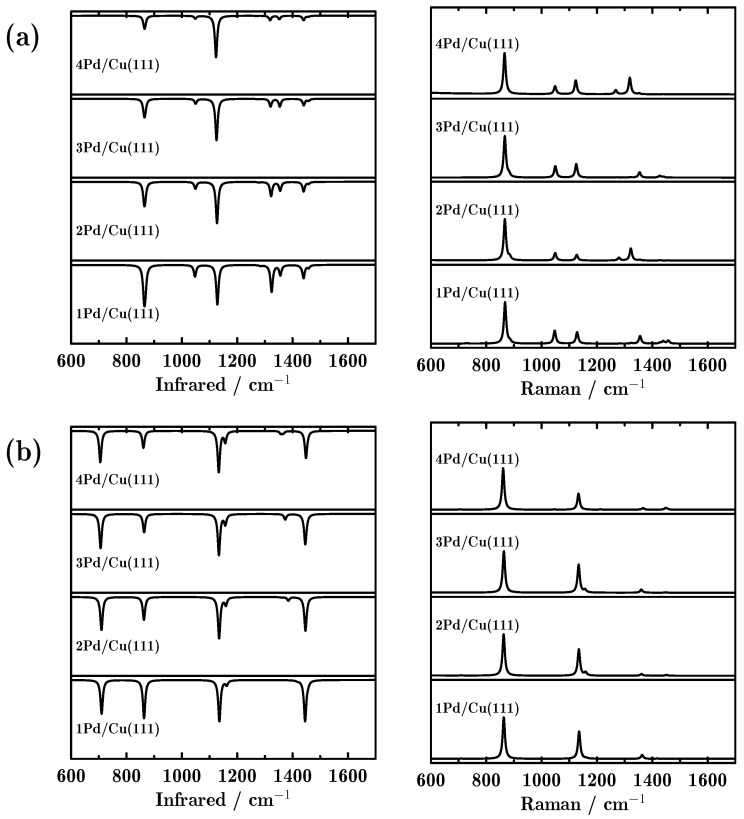
Infrared (IR) adsorption and Raman scattering spectra of propane adsorbed on the Pd-doped Cu(111) surface; (**a**,**b**) indicate two adsorption configurations: perpendicular and parallel adsorbed propane.

In this work, we employed our developed quasi-analytical method [18,19] to study the vibrational spectroscopies of the propane dehydrogenation reaction. Firstly, we evaluate the influence of the number of doped Pd atoms on the vibrational spectra. Figure 2 shows the IR adsorption and Raman scattering spectra for propane_⊥_ and propane_||_ adsorbed on the Pd-doped Cu(111) surfaces. We can see that the peak positions in the IR and Raman spectra were almost independent of the number of doped Pd atoms. For propane_⊥_, the three most intense peaks in the IR spectra were located at 867, 1128 and 1324 cm^−1^, corresponding to C–C–C stretching, methyl CH_3_ rocking and methyl CH_3_ deforming, as shown in Table 2. Interestingly, with more Pd-doped atoms in the surface, the peaks located at around 867 and 1324 cm^−1^ became weaker. For the Raman spectra, there was only one dominant peak located at 867 cm^−1^ for propane_⊥_. Compared to propane_⊥_, propane_||_ showed somewhat different vibrational spectra. To be specific, four dominant peaks were found in the IR spectra, located at around 710, 863, 1136 and 1445 cm^−1^. These four peaks are attributed to methylene CH_2_ rocking, C–C–C stretching, methyl CH_3_ rocking and methyl CH_3_ deforming. Meanwhile, there were only two dominant peaks in the Raman spectra, located at 863 and 1136 cm^−1^. It is noted that for any other intermediate or final products, the IR and Raman spectra varied only a little, no matter how many Pd atoms were doped (all vibrational spectra are provided in Supplementary Information).

**Figure 3:**
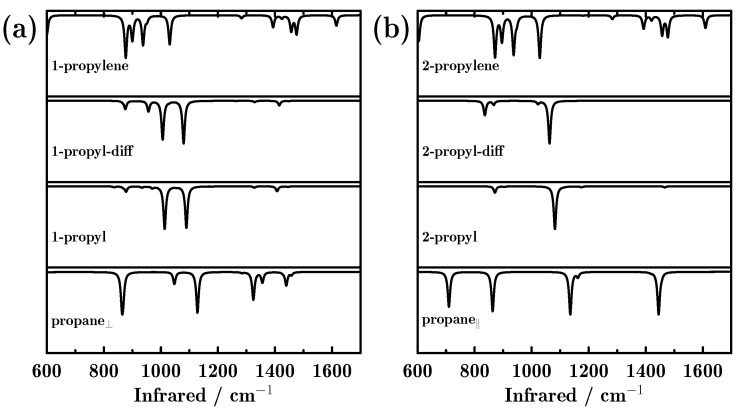
Infrared (IR) adsorption spectra of all reactants, intermediate and final products, during the dehydrogenation of propane towards propylene on the Pd-doped Cu(111) surface; (**a**,**b**) indicate the two reaction paths initiating from parallel and perpendicular adsorbed propane.

Special attention is paid to the change in the vibrational spectra during the reaction process. As shown in Figure 3, the IR spectra changed greatly as the reaction proceeded. Specifically, there were three intense peaks for propane_⊥_, located at 867, 1128 and 1324 cm^−1^. However, after the first hydrogen dehydrogenated from the methyl group, only two dominant peaks (1013 and 1089 cm^−1^) appeared, no matter whether the detached proton was diffused or not. These two peaks are attributed to C–C–C stretching and dehydrogenated methyl twisting. It is noted that due to the dehydrogenation of CH_3_, the symmetry of the adsorbate changed. As a result, the peaks that were related to methyl vanished. When the two dehydrogenations finished and propylene formed, three peaks (877 and 937 and 1030 cm^−1^) dominated the IR spectrum, corresponding to dehydrogenated methylene C–H wagging, dehydrogenated methyl CH_2_ wagging and dehydrogenated methyl CH_2_ rocking. We can see from Figure 3a that the methyl- and methylene-related peaks diminished and the dehydrogenated groups-related peaks appeared.

A similar phenomenon can also be observed for the reaction path initiating from propane_||_. As shown in Figure 3b, four strong peaks dominated the IR spectra for propane_||_. However, after the first hydrogen dehydrogenated from the methylene group, there was only one predominant peak located at 1082 cm^−1^, which is attributed to methyl CH_3_ rocking. The peak located at 710 cm^−1^ (attributed to methylene CH_2_ rocking) diminished because the dehydrogenation occurred on methylene. The IR spectra varied only a little before and after the first detached proton diffused, although the CH_3_ rocking mode showed a 20 cm^−1^ red shift. Finally, the CH_3_ rocking mode become inactive in the IR spectrum after the second H dehydrogenated from the methyl group. It is very interesting to see that the final products obtained from the two reaction paths showed very similar IR spectra, indicating similarity in the geometries.

Several interesting phenomena were observed during the whole catalytic process. For example, the C–C–C stretching mode showed a blue shift, that is, 867, 888, 891 and 937 cm^−1^ for propane_⊥_, 1-propyl, 1-propyl-diff, 1-propylene, and 863, 871, 889, and 937 cm^−1^ for propane_||_, 2-propyl, 2-propyl-diff, and 2-propylene, respectively. On the other hand, the CH_3_ rocking mode showed red shifts during the dehydrogenation. Particularly, 1128, 1114, 1088 and 1031 cm^−1^ were observed for propane_⊥_, 1-propyl, 1-propyl-diff and 1-propylene during the first reaction path. For the second reaction path, these vibrational modes were located at 1136, 1082, 1062, and 1030 cm^−1^ for propane_||_, 2-propyl, 2-propyl-diff, and 2-propylene, respectively.

**Figure 4:**
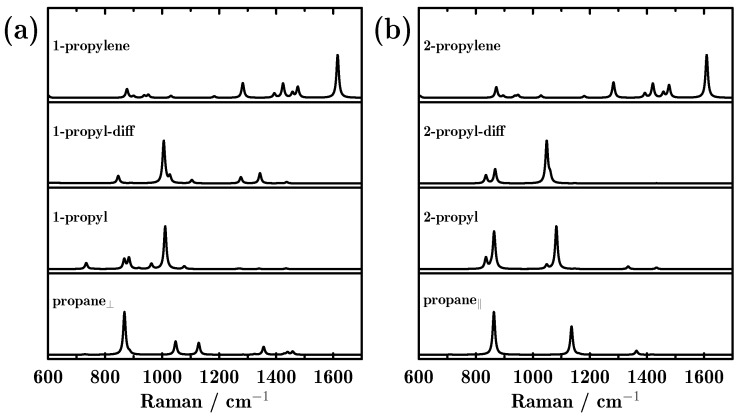
Raman scattering spectra of all intermediate products during the dehydrogenation of propane towards propylene on the Pd-doped Cu(111) surface; (**a**,**b**) represent the two reaction paths initiating from perpendicular and parallel adsorbed propane.

The ability of Raman spectroscopy to monitor reaction processes has also been investigated. As shown in Figure 4, only one peak located at 867 cm^−1^ was found in the Raman spectrum for propane_⊥_, corresponding to C–C–C stretching. After the first proton detached from the methyl group, the most intense peak in the propyl species was located at 1010 cm^−1^, which is attributed to dehydrogenated methyl (–CH_2_) wagging. The diminishing and appearance of Raman peaks revealed the symmetry changes. Additionally, the proton diffusion hardly affected the Raman spectra. Finally, when the second proton dehydrogenated from 1-propyl, only one peak located at 1615 cm^−1^ dominated the Raman spectra, which is attributed to dehydrogenated C=C stretching. For propane_||_, two peaks appeared in the Raman spectra, 863 and 1136 cm^−1^, which are attributed to C–C–C stretching and methyl –CH_3_ rocking. After one proton detached from the methylene group, a small peak split from the main peak. Moreover, a red shift appeared for the –CH_3_ rocking peak, which shifted from 1136 to 1082 cm^−1^. Interestingly, the final product from the two different reaction paths showed similar Raman spectra. From Figure 4, we can also conclude that using Raman spectra, every dehydrogenation step can be easily monitored, and the two different reaction paths can also be distinguished.

## 3. Computational Methods and Details

The spin-polarized DFT calculations were performed using the Vienna ab initio simulation package (VASP) [29,30]. The interactions between the core ions and the valence electrons were described by using the projector augmented wave (PAW) pseudopotentials [31]. The exchange correlation effects were described by the GGA-PBE functional [32]. A plane-wave energy cutoff of 400 eV was used in the calculations. To mimic the flat (111) surface, a four-layer slab of a (5 × 5) unit cell and a 10 Å thick vacuum region were employed here. The Brillouin zone sampling was carried out by using the (5 × 5 × 1) Monkhorst-Pack grids. During the optimization, the lowermost two layers were fixed, while the atoms in the uppermost two layers were allowed to relax until the maximum force became less than 0.02 eV/Å.

After the optimization of the surface system, we fixed all metal atoms and allowed all adsorbate atoms to relax to obtain the normal modes using density functional perturbation theory (DFPT) [33,34]. Then the IR intensity was expressed as

(1) $$I_{\alpha }^{k}=-\sum_{i}\frac{l_{ik}Z_{\alpha i}}{\sqrt{m_{i}}}$$

and the Raman tensors were calculated as

(2) $$R_{\alpha \beta }^{k}=-\sum_{i}\frac{l_{ik}}{\sqrt{m_{i}}}\frac{\partial Z_{\alpha i}}{\partial F_{\beta }}$$

where α and β are Cartesian coordinates, *l* is the Cartesian displacement vector, *m* is the mass of the atom, and $Z_{\alpha i}$ are the Born effective charges, which can be computed analytically. The derivative in Equation (2) is computed by the first-order finite-difference method, that is,

(3) $$\frac{\partial Z_{\alpha i}}{\partial F_{\beta }}=\frac{Z_{\alpha i}\left(\Delta F_{\beta }\right)-Z_{\alpha i}\left(-\Delta F_{\beta }\right)}{2\Delta F_{\beta }}$$

It is noted that the scaling factors are usually needed to make the simulated frequencies more accurate and more comparable to the experimental observations. However, we have noticed that Martin et al. have proven that the scaling factor for the PBE functional is 0.98–0.99 [35]. Furthermore, in the present work, the relative positions and intensities rather than the absolute values played an important role in monitoring the reaction path. As a result, no scaling factor was used in the present work. With the calculated Raman tensor, the differential Raman cross-section could then be calculated using the standard expression [36,37,38]:

(4) $${\left(\frac{d\sigma }{d\Omega }\right)}_{k}=\frac{\pi^{2}}{\epsilon_{0}^{2}}{\left({\tilde{\nu }}_{\mathrm{in}}-{\tilde{\nu }}_{k}\right)}^{4}\frac{h}{8\pi^{2}c{\tilde{\nu }}_{k}}{|{\vec{\varepsilon }}_{i}\cdot \hat{R}\cdot {\vec{\varepsilon }}_{s}|}^{2}\frac{1}{1-\exp\left(-hc{\tilde{\nu }}_{k}/k_{B}T\right)}$$

where $\epsilon_{0}$ is the electric permittivity of free space, ${\tilde{\nu }}_{\mathrm{in}}$ is the wavenumber of incident light (set to be 514 nm), ${\tilde{\nu }}_{k}$ is the wavenumber of the corresponding frequency, *h* is the Planck constant, *c* is the speed of light in a vacuum, $k_{B}$ is the Boltzmann constant, and *T* is the temperature (set to be 298.15 K). Meanwhile, all the calculated Raman cross-sections were convoluted with a Lorentzian function with the full width at half maximum of 15 cm^−1^. Only $R_{zz}^{k}$ needed to be taken into account, in view of the electric field being only along the *z* direction [16], from results based on PBC models.

## 4. Conclusions

In the present work, we propose a method to monitor reaction paths. We systematically studied the IR adsorption and Raman scattering spectra of all reactants, intermediates and final products for the dehydrogenation of propane towards propylene. Two different reaction paths initiating from perpendicular and parallel adsorbed propane were considered. The first-principle calculations of transition-state calculations could hardly distinguish between the two reaction paths. However, using vibrational spectra, every step during the reaction path could be distinguished, and the two different reaction paths could also be easily identified. As a result, the predicted IR and Raman spectra of these reaction intermediates and products provide a basis for their experimental characterization and for the determination of reaction paths.

## Figures and Tables

**Table 1:** Energy barriers for the first C–H bond cleavage from methyl (path 1) and methylene group (path 2) of propane on pure and Pd-doped Cu(111) surfaces [27].

Surfaces	a	b	c	d	e
Path 1 (kcal/mol)	37.9	32.8	31.7	29.9	28.2
Path 2 (kcal/mol)	36.7	31.6	29.6	28.6	26.3

**Table 2:** Calculated infrared (IR) and Raman frequencies assignment of propane_⊥_ (propane_||_), 1-propyl (2-propyl), 1-propyl-diff (2-propyl-diff), and 1-propylene (2-propylene). Only the surface doped by one Pd atom is listed here. Modes 1–7 correspond to methylene –CH_2_– rocking, dehydrogenated methyl –CH_2_ wagging, C–C–C stretching, methylene –CH_2_ wagging, dehydrogenated methyl –CH_2_ twisting, methyl –CH_3_ rocking and methyl –CH_3_ deforming.

Mode	1	2	3	4	5	6	7
Propane_⊥_	729	881	867	1010	—	1128	1324
1-Propyl	745	894	888	1013	1089	1114	1359
1-Propyl-diff	772	889	891	1006	1080	1088	1389
1-Propylene	—	876	937	—	1088	1031	1400
Propane_||_	710	—	863	—	—	1136	1445
2-Propyl	—	—	871	—	—	1082	1443
2-Propyl-diff	—	—	889	—	—	1062	1449
2-Propylene	—	877	937	—	1087	1030	1444

## Supplementary Materials

The supplementary materials are available online.

## References

[1] Zhang R., Zhang Y., Dong Z., Jiang S., Zhang C., Chen L., Zhang L., Liao Y., Aizpurua J., Luo Y. (2013). Chemical Mapping of a Single Molecule by Plasmon-enhanced Raman Scattering. Nature.

[2] Aikens C.M., Madison L.R., Schatz G.C. (2013). Raman spectroscopy: The effect of field gradient on SERS. Nat. Photonics.

[3] Cong S., Yuan Y., Chen Z., Hou J., Yang M., Su Y., Zhang Y., Li L., Li Q., Geng F. (2015). Noble metal-comparable SERS enhancement from semiconducting metal oxides by making oxygen vacancies. Nat. Commun..

[4] Palonpon A.F., Ando J., Yamakoshi H., Dodo K., Sodeoka M., Kawata S., Fujita K. (2013). Raman and SERS microscopy for molecular imaging of live cells. Nat. Protoc..

[5] Pramanik A., Chavva S.R., Nellore V., Priya B., May K., Matthew T., Jones S., Vangara A., Ray P.C. (2017). Development of a SERS Probe for Selective Detection of Healthy Prostate and Malignant Prostate Cancer Cells Using Zn^*II*^. Chem. Asian J..

[6] Jia P., Chang J., Wang J., Zhang P., Cao B., Geng Y., Wang X., Pan K. (2016). Fabrication and Formation Mechanism of Ag Nanoplate-Decorated Nanofiber Mats and Their Application in SERS. Chem. Asian J..

[7] Hu Y., Zhao T., Zhu P., Zhu Y., Liang X., Sun R., Wong C.P. (2016). Tailoring Size and Coverage Density of Silver Nanoparticles on Monodispersed Polymer Spheres as Highly Sensitive SERS Substrates. Chem. Asian J..

[8] Philips D.S., Sreejith S., He T., Menon N.V., Anees P., Mathew J., Sajikumar S., Kang Y., Stuparu M.C., Sun H. (2016). A Three-Photon Active Organic Fluorophore for Deep Tissue Ratiometric Imaging of Intracellular Divalent Zinc. Chem. Asian J..

[9] Auer B., Skinner J. (2008). IR and Raman spectra of liquid water: Theory and interpretation. J. Chem. Phys..

[10] Albrecht A.C. (1961). On the theory of Raman intensities. J. Chem. Phys..

[11] Kurouski D., Zaleski S., Casadio F., Van Duyne R.P., Shah N.C. (2014). Tip-enhanced Raman Spectroscopy (TERS) for in situ Identification of Indigo and Iron Gall Ink on Paper. J. Am. Chem. Soc..

[12] Molesky B.P., Guo Z., Cheshire T.P., Moran A.M. (2016). Perspective: Two-dimensional resonance Raman spectroscopy. J. Chem. Phys..

[13] Girard A., Lermé J., Gehan H., Margueritat J., Mermet A. (2017). Mechanisms of resonant low frequency Raman scattering from metallic nanoparticle Lamb modes. J. Chem. Phys..

[14] Karhánek D., Bučko T., Hafner J. (2010). A Density Functional Study of the Adsorption of Methane-thiol on the (111) Surfaces of the Ni-group Metals: I. Molecular and Dissociative Adsorption. J. Phys. Condens. Matter.

[15] Karhánek D., Bučko T., Hafner J. (2010). A Density-functional Study of the Adsorption of Methane-thiol on the (111) Surfaces of the Ni-group Metals: II. Vibrational Spectroscopy. J. Phys. Condens. Matter.

[16] Zayak A.T., Hu Y.S., Choo H., Bokor J., Cabrini S., Schuck P.J., Neaton J.B. (2011). Chemical Raman Enhancement of Organic Adsorbates on Metal Surfaces. Phys. Rev. Lett..

[17] Ding Z.B., Tommasini M., Maestri M. (2017). First-principles simulation of Raman Spectra of Adsorbates on Metal Surfaces. ChemPlusChem.

[18] Hu W., Duan S., Zhang G., Ma Y., Tian G., Luo Y. (2015). Quasi-Analytical Approach for Modeling of Surface-Enhanced Raman Scattering. J. Phys. Chem. C.

[19] Hu W., Duan S., Luo Y. (2017). Theoretical modeling of surface and tip-enhanced Raman spectroscopies. WIREs Comput. Mol. Sci..

[20] Hu W., Duan S., Zhang Y., Ren H., Jiang J., Luo Y. (2017). Identifying the structure of 4-chlorophenyl isocyanide adsorbed on Au(111) and Pt(111) surfaces by first-principles simulations of Raman spectra. Phys. Chem. Chem. Phys..

[21] Sattler J.J.H.B., Ruiz-Martinez J., Santillan-Jimenez E., Weckhuysen B.M. (2014). Catalytic Dehydrogenation of Light Alkanes on Metals and Metal Oxides. Chem. Rev..

[22] Yang M.L., Zhu Y.A., Zhou X.G., Sui Z.J., Chen D. (2012). First-Principles Calculations of Propane Dehydrogenation over PtSn Catalysts. ACS Catal..

[23] Lo J.M.H., Premji Z.A., Ziegler T., Clark P.D. (2013). First-Principles Investigation of Selective Oxidation of Propane on Clean and Sulfided V_2_O_5_ (010) Surfaces. J. Phys. Chem. C.

[24] Yang M.L., Zhu Y.A., Fan C., Sui Z.J., Chen D., Zhou X.G. (2011). DFT Study of Propane Dehydrogenation on Pt Catalyst: Effects of Step Sites. Phys. Chem. Chem. Phys..

[25] Vajda S., Pellin M.J., Greeley J.P., Marshall C.L., Curtiss L.A., Ballentine G.A., Elam J.W., Catillon-Mucherie S., Redfern P.C., Mehmood F. (2009). Subnanometre Platinum Clusters as Highly Active and Selective Catalysts for the Oxidative Dehydrogenation of Propane. Nat. Mater..

[26] Fu H., Liu Z.P., Li Z.H., Wang W.N., Fan K.N. (2006). Periodic Density Functional Theory Study of Propane Oxidative Dehydrogenation over V_2_O_5_ (001) Surface. J. Am. Chem. Soc..

[27] Cao X. (2016). Insight into mechanism and selectivity of propane dehydrogenation over the Pd-doped Cu(111) surface. RSC Adv..

[28] Henkelman G., Uberuaga B.P., Jónsson H. (2000). A Climbing Image Nudged Elastic Band Method for Finding Saddle Points and Minimum Energy Paths. J. Chem. Phys..

[29] Kresse G., Furthmüller J. (1996). Efficiency of Ab-initio Total Energy Calculations for Metals and Semiconductors Using a Plane-Wave Basis Set. J. Comput. Mater. Sci..

[30] Kresse G., Furthmüller J. (1996). Efficient Iterative Schemes for Ab Initio Total-Energy Calculations Using a Plane-Wave Basis Set. Phys. Rev. B..

[31] Blöchl P.E. (1994). Projector Augmented-Wave Method. Phys. Rev. B.

[32] Perdew J.P., Burke K., Ernzerhof M. (1996). Generalized Gradient Approximation Made Simple. Phys. Rev. Lett..

[33] Gajdoš M., Hummer K., Kresse G., Furthmüller J., Bechstedt F. (2006). Linear Optical Properties in the Projector-augmented Wave Methodology. Phys. Rev. B.

[34] Sternheimer R. (1954). Electronic Polarizabilities of Ions from the Hartree-Fock Wave Functions. Phys. Rev..

[35] Kesharwani M.K., Brauer B., Martin J.M. (2014). Frequency and zero-point vibrational energy scale factors for double-hybrid density functionals (and other selected methods): Can anharmonic force fields be avoided?. J. Phys. Chem. A.

[36] Neugebauer J., Reiher M., Kind C., Hess B.A. (2002). Quantum Chemical Calculation of Vibrational Spectra of Large Molecules—Raman and IR Spectra for Buckminsterfullerene. J. Computat. Chem..

[37] Long D.A. (2002). The Raman effect : A Unified Treatment of the Theory of Raman Scattering by Molecules.

[38] Le Ru E., Etchegoin P. (2008). Principles of Surface-Enhanced Raman Spectroscopy: And Related Plasmonic Effects.

