# Supplementary material for: Monitoring Reaction Paths Using Vibrational Spectroscopies: The Case of the Dehydrogenation of Propane toward Propylene on Pd-Doped Cu(111) Surface
Source: Molecules. 2018 Jan 10;23(1):126. doi: 10.3390/molecules23010126 (PMC6017320; doi:10.3390/molecules23010126)
Supplement: Supplementary file 1 [file molecules-23-00126-s001.pdf]

Supplementary Materials: Monitoring Reaction Paths Using Vibrational Spectroscopies: the Case of Dehydrogenation of Propane toward Propylene on Pd-doped Cu(111) surface

Wei Hu [1] and Xinrui Cao [2*]

S1. IR and Raman Spectra

We have simulated the IR and Raman for all reactants, intermediates and final products. Our studies show that the number of doped Pd atoms hardly influence the vibrational spectra. All obtained IR and Raman spectra are provided herein.

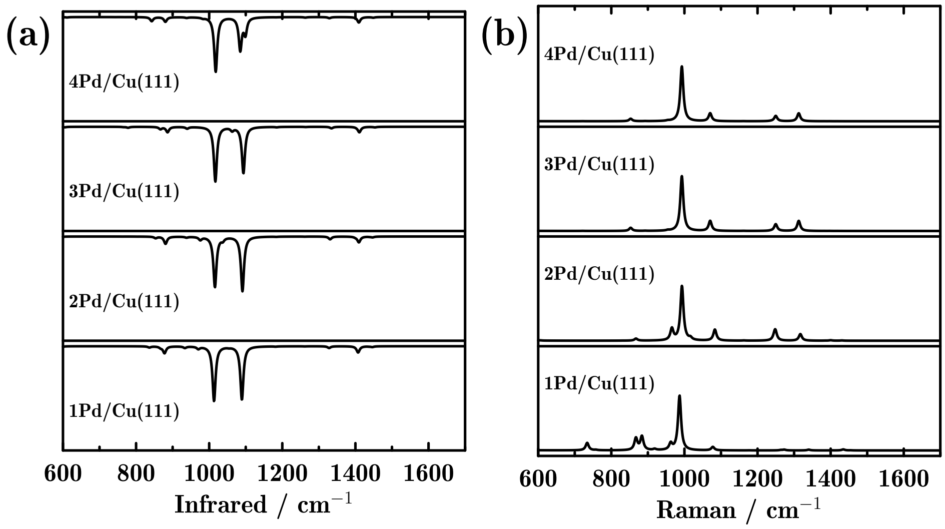

Figure S1. IR (a) and Raman (b) spectra of the intermediate product 1-propyl.

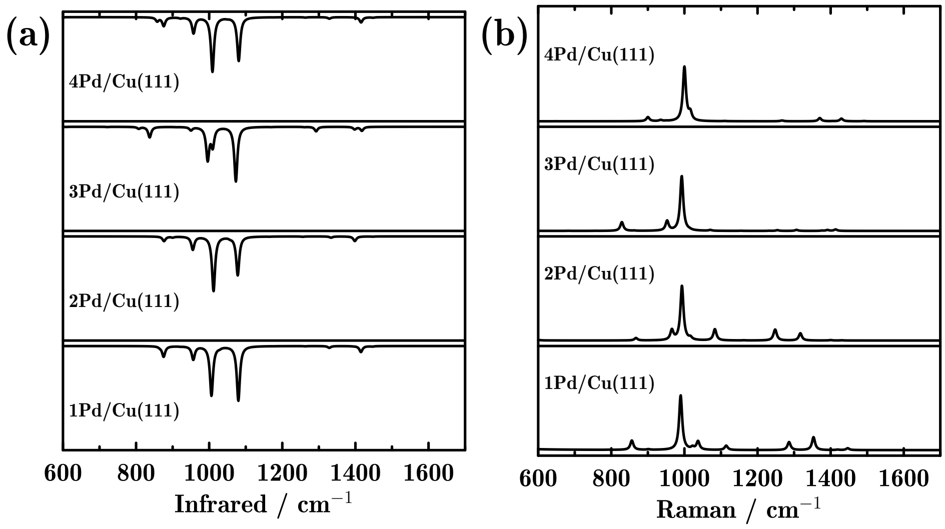

Figure S2. IR (a) and Raman (b) spectra of the intermediate product 1-propyl-diff.

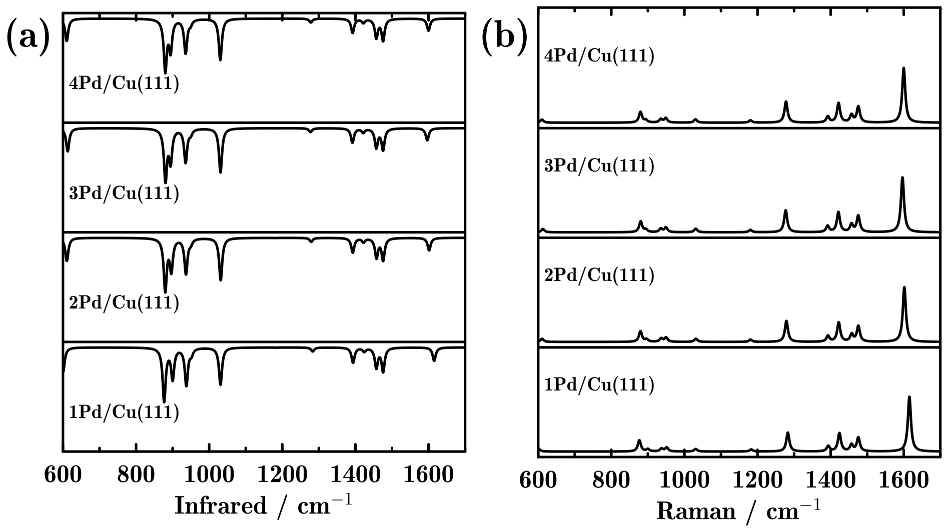

Figure S3. IR (a) and Raman (b) spectra of the final product 1-propylene.

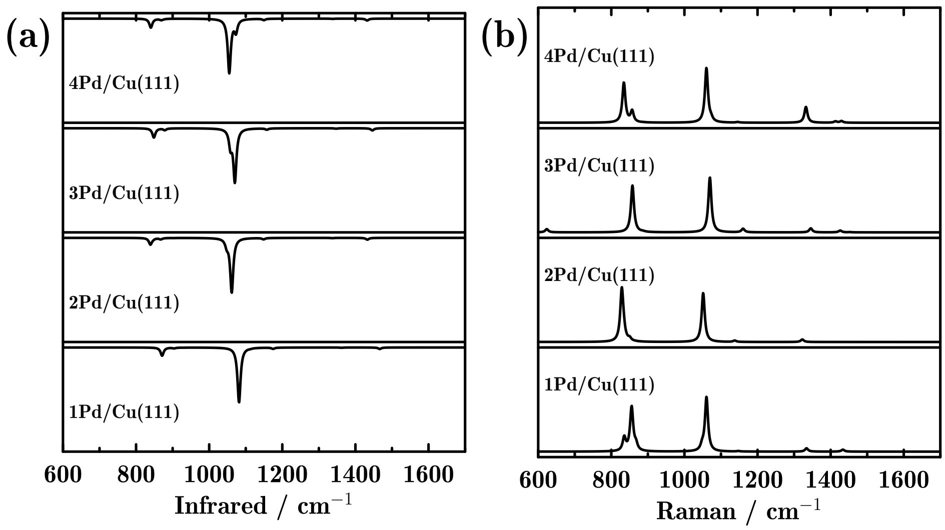

Figure S4. IR (a) and Raman (b) spectra of the intermediate product 2-propyl.

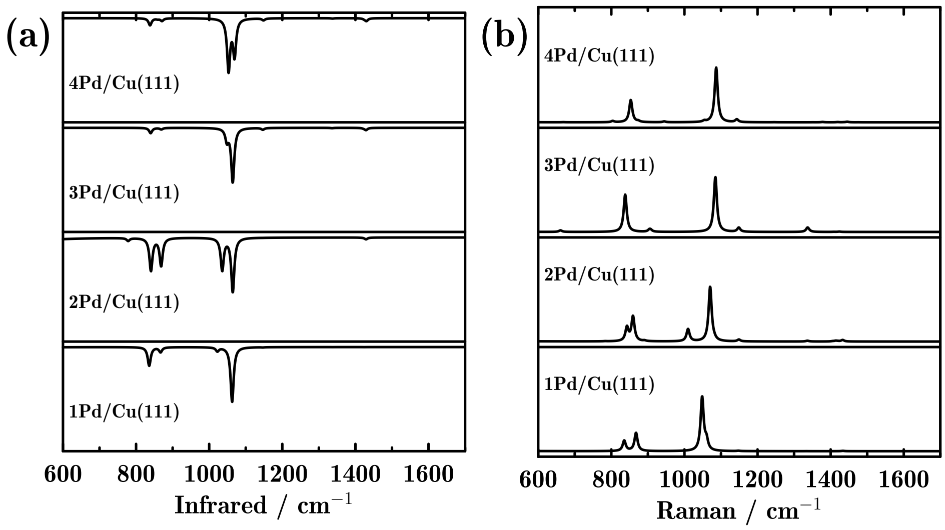

Figure S5. IR (a) and Raman (b) spectra of the intermediate product 2-propyl-diff.

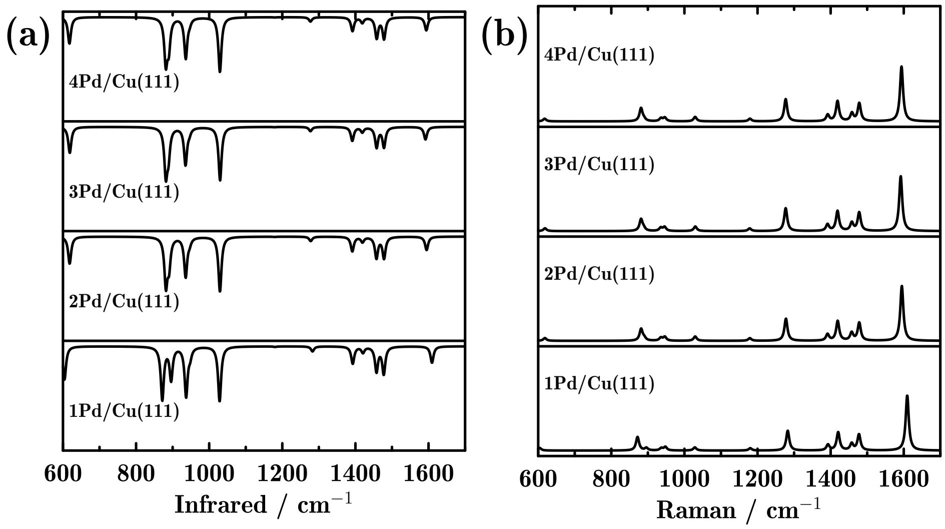

Figure S6. IR (a) and Raman (b) spectra of the final product 2-propylene.
